# The effect of tourniquet application on the morphology and function of quadriceps in patients undergoing total knee arthroplasty: study protocol for a single-blind randomized controlled trial

**DOI:** 10.1186/s13063-022-06114-1

**Published:** 2022-03-05

**Authors:** Ziyang Dong, Yang Li, Liyuan Tao, Hua Tian

**Affiliations:** 1 Department of Orthopedics, Peking University Third Hospital, 49 North Garden Road, Haidian District, Beijing, 100191 China; 2 Engineering Research Center of Bone and Joint Precision Medicine, Ministry of Education, Beijing, China; 3 Research Center of Clinical Epidemiology, Peking University 3rd Hospital, Beijing, China

## Abstract

**Background:**

Tourniquet is currently widely used in total knee arthroplasty to reduce intraoperative blood loss. The academic view of tourniquet application in TKA is now in dispute. Some scholars argue that tourniquet may cause quadriceps injury and bring extra side effects, so they oppose the application of tourniquet. Others find that tourniquet application has no significant adverse impact on TKA patients. Regarding its advantages in reducing intraoperative blood loss, they advocate the regular application of tourniquet in TKA. Quadriceps injury is considered the main cause of tourniquet side effects. There are now many high-quality trials about tourniquet application in TKA but few of them concentrate on quadriceps morphology and function.

**Methods:**

A prospective, single-blind, randomized controlled trial will be adopted. The target sample is 130. Patients who meet the eligibility criteria will be randomly allocated to the tourniquet group and non-tourniquet group. The primary outcome is quadriceps thickness evaluated by ultrasound test. Secondary outcomes include quadriceps stiffness, rehabilitation outcomes, operation time, intraoperative and postoperative blood loss, blood transfusion rate, thigh circumference, VAS score, opioid consumption, d-dimer and C-reactive protein level in the serum, knee function score, postoperative satisfaction score, and complications.

**Discussion:**

This proposed study will contribute to improve evidence of tourniquet application in total knee arthroplasty. This will be a high-quality single-blind randomized controlled trial with a sufficient sample size and strict study design. It will investigate the effects of tourniquet application especially on the morphology and function of quadriceps in patients undergoing total knee arthroplasty and offer advice for tourniquet application in clinical practice.

**Trial registration:**

Chinese Clinical Trial Registry ChiCTR2000035097. Registered on 31 July 2020

## Background

Total knee arthroplasty (TKA) is currently considered the optimal treatment of severe knee osteoarthritis [1, 2]. Intraoperative and perioperative blood loss is quite considerable in TKA because a lot of osteotomy and soft tissue release is required. In order to decrease intraoperative blood loss and create a bloodless surgical field, tourniquet is now widely used in TKA [3, 4]. Its hemostasis function is accomplished by squeezing quadriceps to close lower limb vessels.

However, some scholars argue that tourniquet may have ischemia-reperfusion damage to the quadriceps [5, 6]. They find that tourniquets have no significant impact on decreasing perioperative blood loss but bring certain side effects such as pain, swelling, and deep vein thrombosis for TKA patients [5, 7], so they advocate to reduce or even avoid tourniquet application in TKA. Rames et al. [8] recently published the results from their retrospective study, which showed that patients without a tourniquet had less narcotic consumption and increased distance ambulated prior to discharge. Dong et al. [9] showed less pain and a larger range of motion in patients without a tourniquet. A meta-analysis conducted by Liu et al. [10] also found that the absence of tourniquet could significantly reduce postoperative pain and complication rate and, therefore, be beneficial to Enhanced Recovery After Surgery (ERAS).

In contrast, other scholars hold an opposite idea. Their studies find that tourniquets have no significant impact on postoperative outcomes in TKA patients [4, 11, 12]. Considering the bloodless surgical field and shorter operation time, they propose regular application of tourniquets in TKA. Nicolaiciuc et al. [11] found in a retrospective study that there was no significant difference in pain, narcotic consumption, and range of motion caused by tourniquet application. Furthermore, Jawhar et al. [13] conducted a randomized controlled trial. They introduced the method of a rope pulley isokinetic system and showed that tourniquet had little influence on quadriceps strength and function, patient satisfaction, and physical condition. McCarthy et al. [4] also found there was no significant difference in pain, range of motion, and average length of stay for TKA patients with or without a tourniquet.

Above all, there is no consensus on tourniquet application in TKA, and tourniquet-related side effects are considered relevant to quadriceps injury [14]. Therefore, we design this prospective single-blind randomized controlled trial to focus on tourniquet impact on quadriceps. With the assistance of ultrasonic and rehabilitation tests, we aim to evaluate the tourniquet impact of quadriceps morphology and function for TKA patients, along with operation time, blood management, limb swelling, postoperative pain, inflammatory process, postoperative satisfaction, and complications.

## Methods

### Study design

**Fig. 1:**
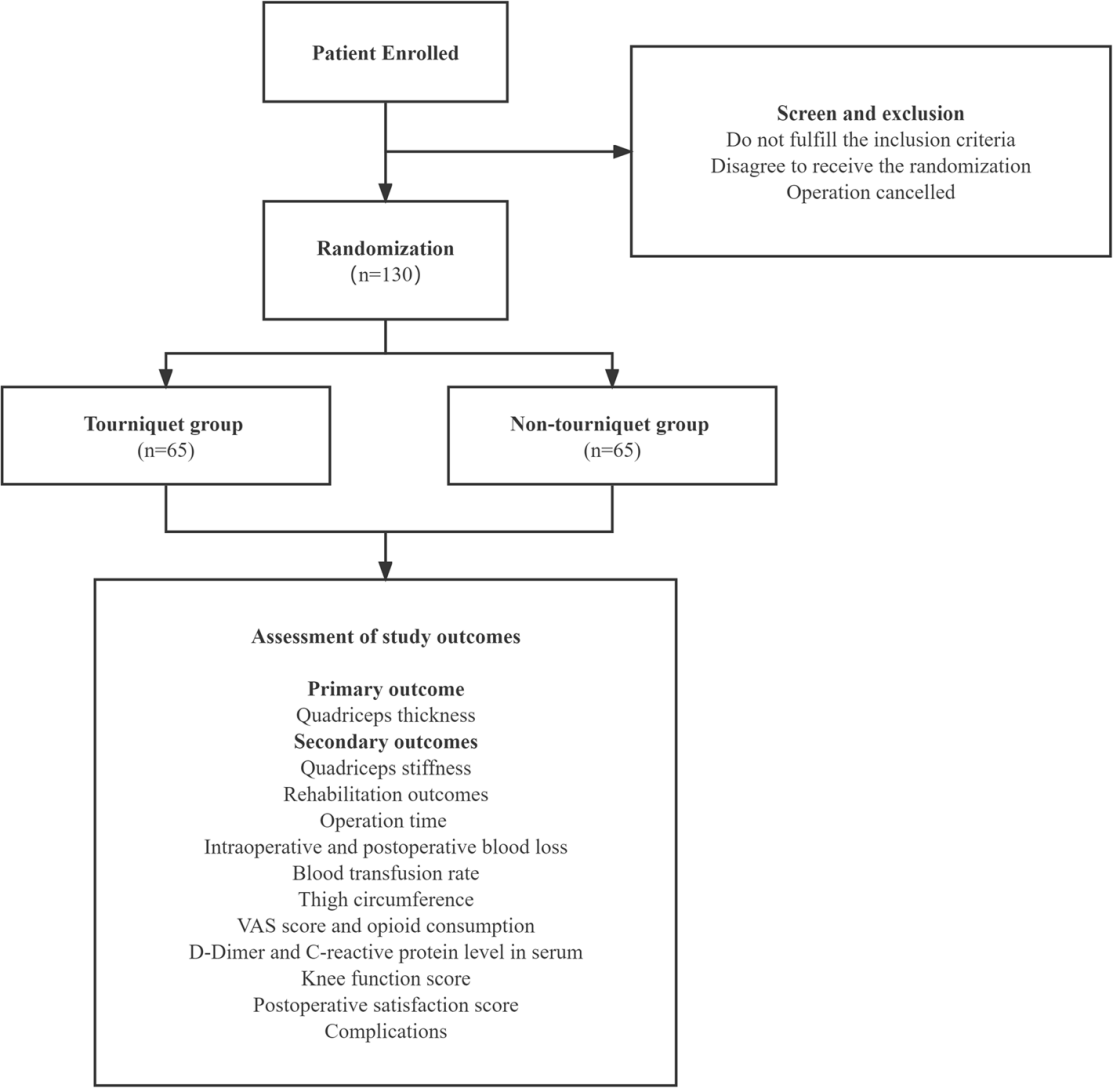
Flowchart of the study process

This study is a clinical randomized controlled trial of estimated 130 patients with knee osteoarthritis undergoing TKA in Peking University Third Hospital (Fig. 1). All patients are randomly divided into the tourniquet group and non-tourniquet group (65 patients each). A tourniquet is applied throughout the surgery in the tourniquet group. In the non-tourniquet group, a tourniquet is tied up but will not be inflated. The trial aims to compare clinical outcomes of TKA with and without a tourniquet. Single binding is performed in this trial which means that the patients are unaware of whether the tourniquets are used in their surgeries. The study will stop once the enrollment is completed.

We assume the primary hypothesis of this trial is that quadriceps thickness in the tourniquet group is not less than that in the non-tourniquet group (non-inferiority study).

### Eligibility criteria and recruitment

The inclusion criteria for this trial are as follows:

1. Patients with primary knee osteoarthritis
2. Planning to operate unilateral primary total knee arthroplasty

The exclusion criteria are as follows:

1. Severe varus (> 25°) or valgus (> 20°) deformity of the knee
2. Extraarticular deformity
3. Past medical history of knee surgery or knee infection
4. Previous long-term consumption of anticoagulant drugs or complicated with the following diseases: renal insufficiency (Cr > 2.5), liver dysfunction, severe heart disease (or coronary stenting in the late 12 months), severe respiratory system diseases, previous venous thromboembolism history or high risk of thrombosis (genetic or acquired thrombosis diseases), blood coagulation dysfunction, the acquired thrombotic diseases, blood coagulation dysfunction, stroke, or malignant tumor history
5. Patients who refuse to sign the informed consent for any reason.

According to the eligibility criteria, a sample of 130 patients is included in this single-center trial. All participants enrolled in this study will receive cost-free postoperative rehabilitation tests and guidance. Post-trial cares such as health consultation and long-term review are also provided.

### Allocation and randomization

All participants who met the study inclusion and exclusion criteria are randomly assigned in a 1:1 ratio to the tourniquet and non-tourniquet groups by the method of random envelope. Prior to study initiation, an independent statistician will use the SAS 9.4 software (SAS Inc., Cary, NC, USA) to generate a blocked random number sequence in permuted blocks of 4 participants. The electronic data capture system automatically generates participant ID numbers in sequence, which are subsequently linked to tourniquet assignments at randomization. Both the participants’ ID numbers and their allocation groups (tourniquet or non-tourniquet) will then be sealed in an envelope. The scientific secretory of this trial is responsible for enrolling participants and assigning participants to intervention (Table 1).

**Table 1:** Schedule of enrollment, interventions, and assessments

Time point		Study period									
		Enrollment	Allocation	Post-allocation							
				t1	t2	t3	t4	t5	t6	t7	t8
Enrollment	Eligibility screen	X									
	Informed consent	X									
	Randomization		X								
Interventions	Tourniquet group				X						
	Non-tourniquet group				X						
Assessments	Demographic data			X							
	Quadriceps thickness			X			X				
	Quadriceps stiffness			X			X				
	Isokinetic muscle strength test								X		
	3D gait analysis								X		
	Posture stability testing								X		
	Thigh circumference			X		X	X				
	VAS score					X	X	X	X		
	Opioid consumption						X				
	Knee function score			X						X	X
	Postoperative satisfaction score									X	X
	Operation time				X						
	Intraoperative blood loss				X						
	Perioperative blood loss						X				
	Blood transfusion rate					X	X	X			
	d-dimer			X		X	X				
	C-reactive protein			X		X	X				
	Complications					X	X	X	X	X	X

*t1* before the operation, *t2* in operation, *t3* 1 day after the operation, *t4* 3 days after the operation, *t5* 2 weeks after the operation, *t6* 6 weeks after the operation, *t7* 3 months after the operation, *t8* 1 year after the operation

Participants and researchers who offer ultrasound and rehabilitation evaluation will all be blinded to the group allocation. However, in case of any medical emergency in the study period, allocation will be unblinded to researchers and clinicians for further medical treatment.

### Trial registration, ethics review, and informed consent

This trial is registered at the Chinese Clinical Trial Registry (registration number: ChiCTR2000035097) and approved by the Peking University Third Hospital Medical Science Research Ethics Committee (Registration number: M2020290).

All the participants sign the informed consent before the trial. An appointed surgeon is responsible for taking informed consent and explain all the intended benefits and risks of tourniquet use to every participant. Consent of participant data and biological specimens are also included in the informed consent. Blood specimens in this study will be disposed after testing and will not be used for genetic analysis or used in other studies. Every participant will be represented by an ID number, and personal information such as name, address, and telephone number will be kept confidential out of Peking University Third Hospital before, during, and after the study. The trial is conducted with the Declaration of Helsinki.

### Baseline procedures

All patients are operated on by the same surgeon. This surgeon has more than 15 years of TKA experience including non-tourniquet TKA and operates at least 300 TKAs per year. All the surgeries are operated by conventional tools. Medial patellar skin incision, medial parapatellar arthrotomy, and measured resection are adopted in all patients. The patella is resurfaced and denervated by hand saw instead of patella replacement. Posterior stabilized implants (Legion from Smith and Nephew, Memphis, TN, USA) are selected.

Perioperative treatments are the same in both groups. Patients all receive intraspinal anesthesia or general anesthesia along with controlled hypotension which is defined as a reduction of the systolic blood pressure to less than 90 mmHg and a reduction of mean arterial pressure (MAP) to less than 65 mmHg or a 30 to 40% reduction of baseline MAP. Before the surgery, all the patients receive a single-time femoral nerve block instead of a continuous femoral nerve block to reduce postoperative pain. Tranexamic acid is used before skin cutting (1 g ivgtt) and before skin suture (1 g ivgtt). Thromboprophylaxis is done with sodium enoxyeparin, 4000 IU per day, starting 12 h after surgery. After the surgery, no femoral block tube or drainage tube is reserved.

Compression of elastic bandage to the surgery limb after suture (removed 24 h after surgery) and active and passive ankle pump training (start immediately after surgery) are adopted in both groups. No patients use continuous passive motion machine. Patients stay in a rest position with the knee in full extension, and they start active and passive rehabilitation training guided by a physical therapist 12–24 h after surgery (local ice compress for 15 min after each training). Patients are encouraged to perform isometric quadriceps exercise and walk with a walker on the first postoperative day. Range of motion (ROM) exercises are performed twice a day in the 6-week period. At 4–6 weeks after surgery, patients are encouraged to walk without aid gait.

The standard plan of postoperative analgesia is also the same in the two groups. Flurbiprofen Axetil (100 mg bid ivgtt) is regularly used for all patients for 2 days after surgery (patients with sulfonamides allergy use parecoxib sodium 40 mg bid ivgtt). If pain control is not satisfying, tramadol or oxycodone (1 tablet once, po) will be used.

### Intervention

A tourniquet is tied to patients in both groups after anesthesia so that tourniquet application is blind to patients. Tourniquet in the tourniquet group is inflated to a standard pressure of (260 ± 10) mmHg (from skin cutting to skin suture). Tourniquet in the non-tourniquet group will not be inflated. There is no concomitant intervention in this study. Proper treatment and care of participants’ combined diseases are allowed. Extra use of analgesic, anticoagulant, and anti-anemia drugs out of the study protocol is prohibited. Participants will be excluded from the trial if these drugs are necessary.

### Adverse event management and emergency termination of the study

Adverse events in this study include, but are not limited to, myocardial infarction, pulmonary embolism, and cerebrovascular disease. Patients will be closely monitored in the study period. If there are suspicions for any adverse event, the study team will discontinue the intervention and break the blind, and patients will be treated appropriately by surgeons and internal medicine physicians. Compensation will be offered to those who suffer harm from trial participation. All adverse events will be recorded and reported in the final manuscript. If patients refuse to participate at any time point during the study, the trial of related patients will be immediately terminated. Reasons of participants who discontinue and deviate from the trial should be recorded.

### Outcomes and measures

#### Preoperative measurements

Demographic information of all the patients is collected, including gender, age, height, weight, and BMI. Baseline outcomes including quadriceps thickness and stiffness, thigh circumference, knee function scores, hematocrit and hemoglobin level, d-dimer, and C-reactive protein level in the serum are evaluated before surgery but after randomization and allocation.

#### Primary outcome

Bilateral lower extremity ultrasonography is performed before and 3 days after surgery to evaluate the thickness of the quadriceps muscle. The inspection is performed by a Canon Apilio I900 ultrasound with L18-5 probe. The probe is placed vertically on the body surface without compressing. The maximum distance from the front to the back of the quadriceps on the horizontal cross-section is selected as the quadriceps thickness; meanwhile, muscle echo and texture are observed. The ultrasound measurements are conducted by the same ultrasound examiner.

#### Secondary outcomes

##### Quadriceps stiffness

Quadriceps stiffness is also evaluated in lower extremity ultrasonography before and 3 days after surgery. With the method of shear wave elastography (SWE), the muscle shear wave propagation velocity (m/s) along the long and short axis in the same section is measured 5 times, and its median is taken as quadriceps stiffness. The occurrence of venous thrombosis is detected simultaneously.

##### Rehabilitation outcomes

It is difficult for end-osteoarthritis patients to accomplish tests of quadriceps function. Therefore, quadriceps function was assessed at 6 weeks postoperatively. Three tests including isokinetic muscle strength testing, three-dimensional gait analysis (3DGA), and posture stability testing are selected.

- Isokinetic muscle strength test

The quadriceps muscle strength is evaluated using BIODEX’s multi-joint isometric training test system. All patients are tested by the same therapist to reduce operator bias in the trial. The instrument shall be systematically calibrated before the test, and the patients shall be informed of the methods and requirements of isometric test, but no warm-up exercises are performed. The non-operative limb is tested first to obtain basic reference data and then the operative limb. Outcomes include peak torque, peak torque/weight ratio, peak torque angle, total work, and average power.

b. Three-dimensional gait analysis

SMART-D 400 3D gait analysis system is used. Patients are asked to make 6 round trips with a natural gait, and the Smart Analyzer software is issued to calculate the spatial and temporal parameters and ankle kinematics parameters. Outcomes include stride time, standing time, swinging time, standing phase, swinging phase, leg support phase, stride length, step length, step speed, stride frequency, and angle change of hip, knee, and ankle when walking.

iii. Posture stability testing

Active Balancer EAB-100 is used for testing. Four static postures in different standing conditions are included: T1, sanding on a hard plate with eyes open; T2, standing on a hard plate with eyes closed; T3, standing on a sponge pad with eyes open; and T4, standing on the sponge with eyes closed. The test time for each condition is 30 s, and there is a rest of 3 min (sitting position) in two test intervals. The real-time movement of the center of plantar pressure is recorded, and the parameters related to its movement are analyzed by professional software. Outcomes include the total track length and average velocity of the displacement of the plantar pressure center.

##### Other secondary outcomes

Other secondary outcomes are also evaluated, including operation time, intraoperative and postoperative blood loss, blood transfusion rate, thigh circumference, VAS score, opioid consumption, d-dimer and C-reactive protein level in the serum, knee function score, postoperative satisfaction score, and complications.

Operation time, which also means tourniquet application time, is evaluated as well. It is defined as the time from skin cutting to the skin suture, and it is accurate to minutes.

Intraoperative blood loss and postoperative blood loss (3 days after surgery) are also evaluated in this trial. Hematocrit level is tested before surgery and 3 days after surgery. Calculating methods are as follows: intraoperative blood loss = intraoperative fluid intake volume + intraoperative irrigation volume + gauze infiltration volume (ml); postoperative blood loss = total blood loss − intraoperative blood loss; total blood loss is calculated by gross linear equation [15]: total blood loss=preoperative blood volume (PBV) ×(preoperative Hct – postoperative Hct)/average Hct, average Hct=(preoperative Hct + postoperative Hct)/2; preoperative blood volume is calculated by Nadler equation [16]: PBV = K1 × height (m)3 + K2× weight (kg) + K3 (for male: K1 = 0.3669, K2 = 0.03219, K3 = 0.6041; for female, K1 = 0.3561, K2 = 0.03308, K3 = 0.1833).

Blood transfusion and transfusion volume after surgery are recorded, and blood transfusion rate is calculated in 1 day, 3 days, and 2 weeks after surgery. Hemoglobin level is tested before surgery as well as 1 day and 3 days after surgery. Blood transfusion is done only if Hb level is lower than 70 g/L, or Hb level is between 70 and 90 g/L with symptoms of anemia like weakness and palpitations. The standard transfusion solution is 400 mL of suspended erythrocyte each time, with a reexamination of blood routine on the second morning.

The circumference of the upper third of the affected thigh is measured with a tape 1 day before surgery as well as 1 day and 3 days after surgery, with an accuracy of 0.1 cm. The swelling degree of quadriceps can be reflected by comparing the circumference in the two groups preoperatively and postoperatively.

VAS scores of the thigh and knee are separately evaluated at 1 day, 3 days, 2 weeks, and 6 weeks after surgery. Opioid consumption (in morphine equivalence) in 3 days after surgery is also recorded to further evaluate postoperative pain.

d-dimer and C-reactive protein (CRP) level in the serum is tested before surgery as well as 1 day and 3 days after surgery to reflect ischemia-reperfusion injury and inflammatory reaction. Complications of patients in both groups such as deep vein thrombosis, hematoma, and anemia are recorded at 2 weeks, 6 weeks, 3 months, and 12 months after surgery.

The knee function score KSS (Knee Society Score) and WOMAC (Western Ontario and McMaster Universities Osteoarthritis Index) are also evaluated before surgery as well as 3 months and 1 year after surgery. Both of the scores are widely used knee function scores worldwide. Their validity and reliability have been verified many times [17–22].

Postoperative satisfaction score is recorded 3 months and 1 year after surgery. It ranges from 1 to 5 points to reflect patients’ satisfaction rate of surgery and recovery. (1 = very dissatisfied, 2 = not satisfied, 3 = neutral, 4 = satisfied, 5 = very satisfied)

In addition, all complications such as deep venous thrombosis, skin vesicle, subcutaneous ecchymosis, and hematoma will be recorded in the study period.

### Follow-up

Participants are followed up via study questionnaires at 2 weeks, 6 weeks, 3 months, and 12 months after surgery. In 2-week follow-up, the wound recovery, blood transfusion volume, and VAS score of the affected limb are recorded. In a 6-week follow-up, quadriceps function is evaluated by isokinetic muscle strength testing, three-dimensional gait analysis, and posture stability testing. KSS, WOMAC, and postoperative satisfaction score are recorded 3 and 12 months after surgery. Complications are recorded in every follow-up.

### Data collection and management

All the data should be collected timely, correctly, and completely in the case report form. The stiffness and thickness of the quadriceps muscle are collected by ultrasound researchers. The isokinetic muscle strength tests, three-dimensional gait test, and balanced postures test are collected by rehabilitation researchers. The rest of the outcomes are collected by orthopedic researchers. All the data is stored in the database and processed by Microsoft Excel®.

The data management committee consisting of orthopedic surgeons, statisticians, and administrative staff is established to assess the progress of this study, so as to ensure the safety of study participants and to maintain the study’s scientific integrity.

### Auditing and amendments

To guarantee the quality of trial conduction, experts independent of this trial will be invited to audit the trial implementation process every 2 months. The experts may have access to source documents and study records to ensure the quality of trial conduct. There is no interim analysis in this study. Major amendments to this study protocol will be communicated with investigators, trial regulators, journals, and the ethics committee.

### Statistical analysis

The statistician who conducted the analysis is blinded to the group allocation. Summary statistics are used to describe the participant characteristics of the trial groups at baseline in the intention-to-treat (ITT) analysis set. The missing data of quadriceps thickness and stiffness is imputed by the multiple imputation method. The results of multiple imputation data are used as a type of sensitivity analysis for comparing primary outcomes between the groups.

For primary outcomes comparison, the ITT analysis is performed to evaluate the differences between the groups, and effective analysis population (EAP) and per-protocol (PP) analyses are also performed for sensitivity assessment. The primary outcomes do not follow the Gaussian distribution and are presented as median (IQR) and tested by the Mann-Whitney *U* test. Bonferroni correction is used to reduce the significance level. The means (95% CIs) of the between-group differences of the medians are calculated by the bootstrap method (1000 replications). The generalized linear mixed models (GLMM) are also performed for the primary endpoint, including group, gender, age, height, and weight as fixed covariates.

For comparing the secondary and exploratory endpoints, continuous data are presented as means (SDs) or medians (IQRs) as appropriate. The secondary endpoints are analyzed by the linear mixed model (LMM), adjusted for gender, age, height, weight, and other covariates. The correlation type of different measurement time points is assumed as the first-order autocorrelation in the LMM. Exploratory endpoints are compared by the Mann-Whitney *U* test, and the significance level is submitted to Bonferroni correction.

For safety endpoints, categorical data are presented as counts and percentages and tested by the Pearson’s chi-square test or Fisher’s exact test. The 95% CIs of absolute risk differences between the groups are calculated by the Newcombe-Wilson Score method. All statistical analyses are conducted with the statistical package SPSS, V.18.0 (SPSS Inc.) and the R 3.4.0 software. Besides Bonferroni correction, statistical significance is defined as *p* < 0.05with two-sided testing.

### Pilot study

A pilot study has already been conducted. We enrolled 10 knees with primary osteoarthritis undergoing. Five knees were operated with a tourniquet, and the other 5 knees were operated without a tourniquet. Postoperative swelling, VAS score, quadriceps morphology, and function were recorded. Finally, the pilot study found that there was no significant difference in these outcomes between the two sides, indicating that a tourniquet might not cause severe quadriceps injury.

### Sample size estimation

The primary hypothesis of this trial based on our previous pilot study is that quadriceps thickness in the tourniquet group is not less than that in the non-tourniquet group. The risk of class I error of the hypothesis is controlled within 5% during sample size estimation (*α* = 0.05).

Quadriceps thickness is 3.1 ± 0.5 cm in the non-tourniquet group and 3.1 ± 0.6 cm in the tourniquet group. Non-inferiority margin (*σ*) is 0.31, which is 10% of the average quadriceps thickness in the non-tourniquet group, *α* = 0.025 (unilateral), *β* = 0.2. We apply the non-inferiority test by PASS14 and find that we need at least 51 participants in each group. We determine that a total of 51 participants per group would have an 80% statistical power in detecting a tourniquet impact between the two groups. This will result in a total of 102 participants. Estimating that 30% of participants would drop out, a sample size of 130 participants is considered to be adequate for this study.

### Dissemination plan

The results of the trial will be submitted to an international peer-reviewed journal. The results will also be presented at national and international conferences relevant to the subject fields. Researchers who have a contribution to this study will be listed in the authorship or acknowledgements. The full protocol, participant-level dataset, and statistical code are granted public access.

## Discussion

Tourniquet is now widely used in TKA, but its latent adverse effects are problems that we cannot neglect. Some researchers recommend regular tourniquet application in TKA because of its advantage in reducing intraoperative blood loss and operation time. Other researchers hold an opposite idea. They believe that tourniquet application has obvious damage to quadriceps and extra side effects such as pain, swelling, and blood loss, which may go against patients’ Enhanced Recovery After Surgery (ERAS), so they resist using a tourniquet in TKA. After years of studies including meta-analysis and randomized controlled trials, tourniquet application in TKA is still in dispute.

Tourniquet effects on quadriceps morphology and function are controversial. It is generally considered that tourniquet does have certain damage to the quadriceps, but how bad it can be and what effects it will have on patients are confusing. Leurcharusmee et al. [6] hold the point that tourniquet application can cause ischemia-reperfusion injury to quadriceps, which leads to the release of both oxygen free radicals and inflammatory cytokines. Dennis et al. [23] found in a prospective trial that tourniquet application can reduce quadriceps strength in 3 months after surgery. Guler et al. [24] found that tourniquet use in TKA can decrease the thigh and quadriceps muscle volumes. However, Jawhar et al. [13] reported that tourniquet has no significant effects on quadriceps strength and function. Ayik et al. [25] also reported that avoiding tourniquet cannot improve quadriceps strength after TKA.

It is generally considered that tourniquet application can significantly reduce intraoperative blood loss, but its effects on total blood loss are not explicit. Zhao et al. [15] found out in a randomized controlled trial that tourniquet application may increase hidden blood loss and total blood loss. However, Liu et al. [10] conducted a meta-analysis and found that tourniquet has no significant effects on total blood loss. Herndon et al. [3] hold another idea that tourniquet may reduce total blood loss but has no effects on postoperative blood transfusion rate.

Postoperative pain is an important outcome for TKA patients. It can be evaluated by VAS score as well as opioid consumption. Tourniquet effects on postoperative pain are also controversial. Liu et al. [7] hold the point that tourniquet application can increase postoperative pain, which may be caused by ischemia-reperfusion injury and the release of cytokines. Ajnin et al. [16] and Dong et al. [9] both reported that not using tourniquet in TKA can reduce the patient’s postoperative pain and be beneficial to their recovery. While a randomized controlled trial conducted by McCarthy et al. [4] reported that tourniquet application had no significant effect on thigh pain. Palanne et al. [26] also reported that tourniquet application could not affect pain management and postoperative opioid consumption.

Lower limb swelling is also related to postoperative recovery. It can be measured by thigh circumference. Tourniquet effects are still disputable in respect of swelling. Ajnin et al. [16] and Wang et al. [27] both reported that patients without tourniquet application showed less lower limb swelling. However, Alexandersson et al. [28] reported that tourniquet application had no significant effects on limb swelling.

Postoperative inflammation process is also in association with tourniquet application. It can be reflected by the CRP level in the blood. A randomized controlled trial conducted by Cao [29] reported an increase in inflammatory mediators in tourniquet application such as TNF-α, PTX3, CCL2, PGE-2, and SOD-1, and its degree of elevation is positively correlated with the tourniquet time, which is considered relative to ischemia-reperfusion injury caused by a tourniquet. Zhao et al. [15] also reported that the absence of tourniquet application could reduce the postoperative inflammation process.

Above all, there is still no consensus on tourniquet effects on quadriceps morphology and function, blood loss, postoperative pain, lower limb swelling, and other outcomes for patients undergoing TKA. Tourniquet-related side effects are mainly about quadriceps injuries, so we decide to concentrate ourselves on tourniquet effects on quadriceps morphology and function and design this single-blind randomized controlled trial to offer advice for tourniquet application in clinical practice.

There is now little study about quadriceps morphology of patients after TKA. Quadriceps thickness is selected as the primary outcome to reflect quadriceps morphology, which is measured by an ultrasound test. Innovatively, we use shear wave elastography (SWE) to evaluate quadriceps stiffness. SWE is an evolving ultrasound technique which is progressively used in the musculoskeletal system to evaluate tissue elasticity [30–32]. It is reported to evaluate the stiffness of soft tissues such as quadriceps tendon or medial collateral ligament in healthy people [33, 34], but it has never been used to assess quadriceps stiffness of patients undergoing TKA. Shear waves propagate faster through stiffer tissue so we decide to measure shear wave velocity along the long and short axes of quadriceps to represent quadriceps stiffness [35, 36]. Additionally, we include three tests of rehabilitation department. The isokinetic muscle strength test has been proved to be valid in the assessment of muscle strength in TKA patients [37, 38]. Three-dimensional gait analysis is a useful clinical test to evaluate gait abnormality which can be captured by cameras placed around a walkway [39]. Posture stability testing is also used in our study to monitor the displacement of the plantar pressure center [40]. These 3 tests can provide important indicators to evaluate quadriceps function for TKA patients.

Other outcomes including intraoperative and postoperative blood loss, blood transfusion rate, thigh circumference, VAS score, opioid consumption, d-dimer and C-reactive protein level in the serum, knee function score, postoperative satisfaction score, and complications in the tourniquet group and non-tourniquet group will be evaluated. These outcomes work as effective supplements to our study to further evaluate tourniquet effects on TKA patients.

What’s more, tourniquet time is also a meaningful indicator. As tourniquet is used throughout the whole operation period, it can be represented by operation time. Quadriceps injury and many other side effects may be time-related, so we hope to further investigate the relationship between tourniquet time and tourniquet effects in this study.

Indeed, there are several limitations to our study. First, an ultrasound test is examiner-dependent. Though all the patients are tested by the same ultrasound examiner, it may still affect the validity of the assessment to some degree. Second, tourniquet effects on patients may be too subtle to be reflected by chosen outcomes like VAS score or knee function score. More sensitive outcomes for patients after TKA are needed. Third, this study mainly focuses on clinical outcomes of tourniquet effects. There is a lack of deeper study of the mechanism of ischemia-reperfusion and inflammation.

This study is precisely designed to clarify the effect of tourniquet application on the morphology and function of quadriceps in patients undergoing total knee arthroplasty and offer advice for tourniquet use in clinical practice. Indeed, tourniquet application cannot be determined by one single trial, but we believe that more high-quality studies will be conducted and tourniquet impact will be clarified in the foreseeable future.

## Trial status

Recruitment commencing July 2020. Eighty-four participants have already been recruited. Important protocol amendments will be communicated to relevant bodies. Recruitment will be finished at the end of 2022.

## Data Availability

The datasets during and/or analyzed during the current study are available from the corresponding author on reasonable request.

## Abbreviations

TKA: Total knee arthroplasty
VAS: Visual analog scale
SWE: Shear wave elastography
CRP: C-reactive protein
KSS: Knee Society Score
WOMAC: Western Ontario and McMaster Universities Osteoarthritis Index
